#  A Pulse-Biasing Small-Signal Measurement Technique Enabling 40 MHz Operation of Vertical Organic Transistors

**DOI:** 10.1038/s41598-018-26008-0

**Published:** 2018-05-16

**Authors:** Bahman Kheradmand-Boroujeni, Markus P. Klinger, Axel Fischer, Hans Kleemann, Karl Leo, Frank Ellinger

**Affiliations:** 1 Chair for Circuit Design and Network Theory (CCN), Technische Universität Dresden, Helmholtzstr. 18, 01069 Dresden, Germany; 2 Center for Advancing Electronics Dresden (cfaed), Technische Universität Dresden, Würzburgerstr. 46, 01187 Dresden, Germany; 3 Dresden Integrated Center for Applied Physics and Photonic Materials (IAPP), Technische Universität Dresden, Nöthnitzerstr. 61, 01187 Dresden, Germany

## Abstract

Organic/polymer transistors can enable the fabrication of large-area flexible circuits. However, these devices are inherently temperature sensitive due to the strong temperature dependence of charge carrier mobility, suffer from low thermal conductivity of plastic substrates, and are slow due to the low mobility and long channel length (L). Here we report a new, advanced characterization circuit that within around ten microseconds simultaneously applies an accurate large-signal pulse bias and a small-signal sinusoidal excitation to the transistor and measures many high-frequency parameters. This significantly reduces the self-heating and therefore provides data at a known junction temperature more accurate for fitting model parameters to the results, enables small-signal characterization over >10 times wider bias I–V range, with ~10^5^ times less bias-stress effects. Fully thermally-evaporated vertical permeable-base transistors with physical L = 200 nm fabricated using C_60_ fullerene semiconductor are characterized. Intrinsic gain up to 35 dB, and record transit frequency (unity current-gain cutoff frequency, f_T_) of 40 MHz at 8.6 V are achieved. Interestingly, no saturation in f_T_ − I and transconductance (g_m_ − I) is observed at high currents. This paves the way for the integration of high-frequency functionalities into organic circuits, such as long-distance wireless communication and switching power converters.

## Introduction

Organic electronics is expected to provide a technology platform for emerging applications which require large-area mechanically-flexible light-weight circuits. For instance, organic light-emitting devices have been demonstrated on plastic substrate for flexible and unbreakable displays^1^. Furthermore, organic devices can be made ultra-thin^2,3^, stretchable^4^, and can conformally interface with the skin and moving internal organs such as uneven heart tissue^5^, making them suitable for health monitoring and artificial electronic skins^6^. The use of organic transistors has also been demonstrated for ferroelectric memories^7^ and sophisticated electrical circuits. E.g. using inductively-coupled coil antennas, organic RFID tags can be employed for short distance (2–5 cm) wireless communication^8^, or a fully-printed bendable audio system has been demonstrated^9^, consisting of a 128 cm^2^ piezo-polymer loudspeaker and a self-biased organic audio amplifier.

While compact device modeling is a key requirement for complex circuit simulation and system design, accurate device characterization and parameter extraction is a prerequisite for fine tuning and optimization of model parameters. In this regard, the characterization and modeling of organic transistors is for two reasons particularly difficult: Firstly, there are unusual physical effects in organic transistors that cause serious difficulties when extracting static modeling parameters such as charge carrier mobility or gate-source capacitance. For example, when using conventional long-channel Field-Effect Transistor (FET) equations for calculating the charge carrier mobility, the presence of a kink in the transfer I–V characteristic can cause significant mobility overestimation^10,11^; large contact or source/drain electrode resistances result in large mobility underestimation^12,13^; or measuring the gate capacitance at high frequencies and using this value for extracting the mobility from DC, i.e. near 0 Hz, I–V measurements brings an additional error if there is a strong gate capacitance frequency dependence^13^.

Secondly, there are also time-dependent processes happening in organic transistors such as self-heating and bias-stress so that the dynamic behavior of organic FETs is insufficiently described by state-of-the-art DC parameter models.

Self-heating is an important physical effect which occurs during the device characterization at high DC I× V points and has two major drawbacks. First, it results in an unknown device junction temperature if the junction-to-substrate thermal resistance is not known, making the measured data unsuitable for fitting any compact models into it. Second, junction burning or breakdown limits the maximum I–V point at which the device can be characterized, whereas in some real circuits such as digital logic gates and super regenerative oscillators only a very high transient pulse current is passing through the device during the switching or on time.

Self-heating is an issue in all semiconductor-based devices, but organic transistors are inherently more sensitive to it because their charge carrier mobility strongly increases with temperature^14,15^, and their source/drain metal electrode to polymer channel contact resistance rapidly decreases with it^15^. In addition, these devices are mostly intended for flexible plastic substrates, which are in general very poor thermal conductors resulting in large junction to substrate thermal resistance. This problem is partly alleviated by low current densities in organic devices. However, these devices generally work instead at relatively higher voltage levels, and the current density will increase by development of higher mobility organic semiconductors in the future.

Device measurement can be divided into two general domains of large-signal and small-signal measurements. For the large-signal I–V characterization, the self-heating can be suppressed by applying short pulses instead of long-time DC biasing^14^. This can be easily done using existing commercial equipment such as the Keysight Precision Source/Measure Unit B2912A. However, for the case of small-signal measurements this is absolutely not straightforward and has not been done so far. All existing small-signal measurement techniques work based on the principle of long-time DC biasing of the device at a known I–V point, and then superimposing a known small-signal voltage/current on this DC bias at one input terminal of the device, and measuring the output small-signal voltage/current at another terminal of the device. For example, this approach has been used in our previous works^13,16^ for measuring the transconductance (g_m_), intrinsic gain (A_v0_), gate/base impedance, and current gain (h_21_); for S-parameter measurement^17^; and for transit frequency (f_T_) measurement^18–20^.

In this context, DC biasing of the device also has a third drawback. Most organic transistors still suffer from the bias-stress effect, which is due to the trapping of charge carries, and causes dynamic shift of the threshold voltage as well as the electric field inside the device^21,22^. Therefore, applying a DC bias during long-time measurements changes the characteristics of the device and reduces the accuracy and repeatability of the results.

In order to overcome the above-mentioned problems, we present in this paper for the first time an advanced pulse-biasing characterization circuit, which can turn on the organic transistor and apply an accurate bias I–V to it in less than ten microseconds, then apply a small sinusoidal signal to the device and measure several small-signal parameters such as h_21_, f_T_, g_m_ and A_v0_, and then quickly turn off the device again. This approach significantly reduces the junction temperature increase, and allows measurements at much higher bias currents. In addition, the new setup can be used for measuring the temporal evolution of stress and self-heating effects over a μs to ms time scale.

**Figure 1:**
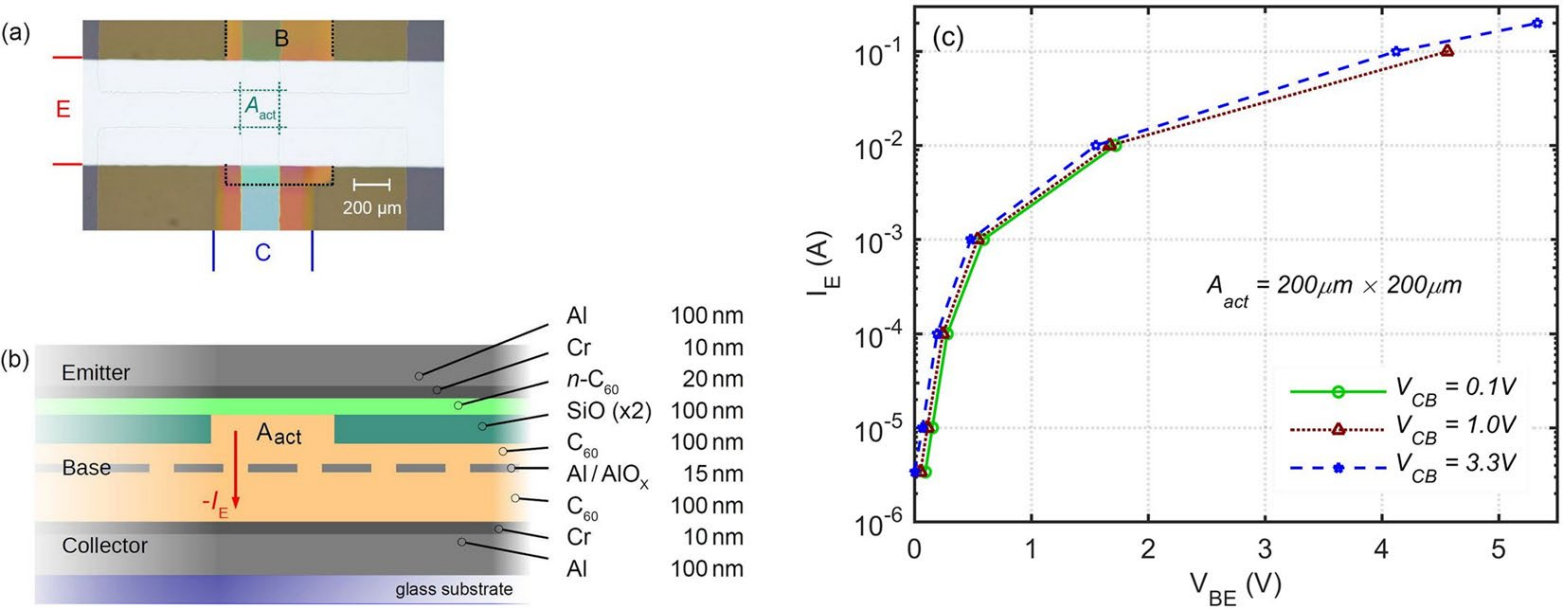
Organic permeable base transistor (OPBT). (**a**) Modified device photo showing the three electrodes and the active area from top view; dot lines are added around the base and A_act_ (**b**) Drawn device structure, side view in A_act_. (**c**) Emitter current as a function of base-to-emitter voltage.

As a case study, we characterized the fully-thermally-evaporated n-type vertical Organic Permeable-Base Transistor (OPBT)^23^, shown in Fig. 1. The OPBT is a promising low-voltage high-current high-speed device, fabricated solely using low-resolution low-cost shadow masks. This transistor resembles triodes and bipolar junction transistors, but here we have a thin metal base layer that contains naturally occurring pores. The native aluminum oxide of this electrode leads to a channel formation around it and the fact that the base potential controls the electrons flowing from emitter to collector. Space charge limited current (SCLC) in the undoped C_60_ fullerene layers is the main limitation of this transistor^24^, whereas electron injection is very efficient due to the thin n-doped C_60_ layer. Details of the device fabrication and operation mechanism, DC I–V characteristics, SCLC and device simulation can be found in other publications^23,24^, as well as the methods section.

This manuscript is organized in the following way. Firstly, we demonstrate the pulse-biasing measurement results of an OPBT device including the small-signal performance, and the temporal evolution of the charge distribution and self-heating effects occurring in the device. Secondly, we focus on the characterization circuit and small-signal parameter extraction techniques. Finally, we discuss the consequences of our findings on the context of real circuit applications.

## Case Study Measurement Results

Measurements are performed at a controlled room temperature of ~25 °C, on the samples shown in Fig. 1, with an active area of Aact = 200 μm × 200 μm, at three low/moderate/high collector-to-base voltages of VCB = 0.1 V/1.0 V/3.3 V, respectively.

Transfer large-signal pulse I–V characteristics of the OPBT are shown in Fig. 1(c), reaching a peak current of I_E_ = 200 mA at a total voltage of V_CE_ = V_CB_ + V_BE_ = 8.6 V. This corresponds to a current density of 5 μA/μm^2^ in the A_act_. At V_CE_ = 1.0 V, the device can still drive 47 nA/μm^2^.

### Transit Frequency (f_T_)

The small-signal current gain (h_21_) is defined as the ratio of the small-signal collector current (i_c_) over the small-signal base current (i_b_) measured when both emitter and collector are small-signal AC ground.

**Figure 2:**
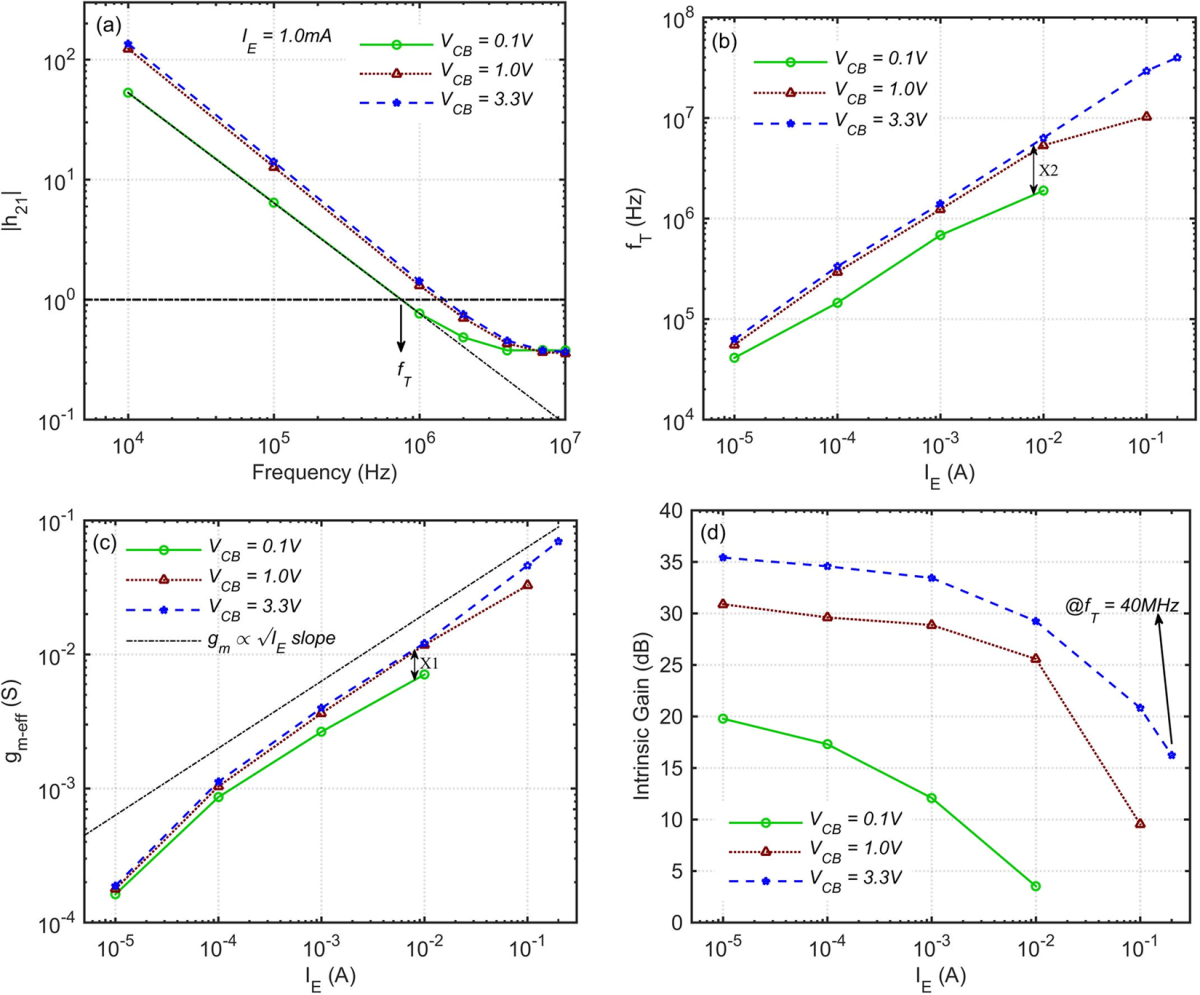
Measured small-signal performance of the OPBT with A_act_ = 200 μm × 200 μm. (**a**) Current gain at 1 mA pulse bias versus frequency. (**b**) Transit frequency versus pulse-biased emitter current. (**c**) Transconductance versus pulse-biased emitter current. (**d**) Intrinsic voltage gain A_v0_ versus pulse-biased emitter current.

An example of the magnitude of h_21_ as a function of frequency at a pulse bias I_E_ = 1.0 mA at three different V_CB_ is shown in Fig. 2(a). The h_21_ is decreasing ~20 dB per decade (i.e. proportional to 1/f) as known for conventional transistors. f_T_ is defined as the frequency at which the extrapolation from the low frequency part of h_21_ falls to unity. It is an important figure of merit of a transistor, indicating the frequency range in which the device can amplify the input current signal.

Measured f_T_ versus pulse bias emitter current is shown in Fig. 2(b), reaching f_T_ = 40 MHz at pulse I_E_ = 200 mA; i.e. current density of 5 μA/μm^2^; at V_CB_ = 3.3 V, V_BE_ = 5.3 V and V_CE_ = 8.6 V; measured at the shortest possible pulse duration of ~10 μs. To the best of our knowledge, this is the highest measured f_T_ for an organic transistor so far. As a comparison, f_T_ = 27.7 MHz at around three times higher voltage of V_DS_ = 25 V for C_60_, and 11.4 MHz for Pentacene at 25 V were previously reported for planar transistors on glass substrate^19^, fabricated using high-resolution patterning techniques such as photolithography and lift-off process. f_T_ = 20 MHz at 30 V was achieved using laser sintering of high resolution electrodes on glass^18^. In vertical structures, f_T_ = 1.5 MHz was reported for step edge devices^25^, and f_T_ = 20 MHz at 15 V for a 3D transistor structure^20^.

### Transconductance (g_m_)

The effective transconductance versus pulse bias current is shown in Fig. 2(c) and is calculated as the ratio of the i_c_ to the small-signal base-emitter voltage; measured below the frequency of f_T_/10. Interestingly, no saturation of the g_m-eff_ at high current densities is observed. This indicates that the parasitic emitter resistance R_E_ is small, otherwise g_m-eff_ = g_m_/(1 + g_m_ × R_E_) would eventually saturate to 1/R_E_ at high currents. The small R_E_ means that the electrode resistance is small and the n-C_60_ doped layer is making low impedance interfaces to both Cr and intrinsic-C_60_ layers.

Considering the equation f_T_ ≈ g_m_/(2π × (C_be_ + C_bc_)), increasing the V_CB_ bias from 0.1 V to 3.3 V at fixed I_E_ in Fig. 2(c) improves the transconductance, however, the amount of the corresponding f_T_ or h_21_ improvement at the same I_E_ in Fig. 2(b or a) is always higher, e.g. X2 is 89% more than X1. This is because of the fact that increasing the V_CB_ depletes the collector-base C_60_ layer from the charge carriers, and this also reduces the C_bc_ and therefore further improves the f_T_.

### Intrinsic Gain (A_v0_)

Intrinsic gain is the maximum small-signal voltage gain that a device can provide, and is equal to g_m_ × r_out_, where r_out_ = ∂V_CE_/∂I_C_ is the output resistance of the device. Basically a transistor with A_v0_ less than one, i.e. 0 dB, is a useless device, because it cannot perform any amplification.

In general, A_v0_ decreases with scaling down the channel length. However, as shown in Fig. 2(d), OPBT can provide a good gain of 35 dB at low currents and a fairly acceptable gain of 16 dB at the f_T_ = 40 MHz bias point, i.e. I_E_ = 200 mA, with a short physical L = 200 nm. With A_v0_ = 16 dB, one can make an amplifier with a gain of 10 dB per stage by means of the bootstrapping technique^16^.

A diffusion-driven organic transistor was recently reported^26^, which can even provide 57 dB of intrinsic gain at W/L = 100 μm/12.5 μm. However, this transistor can drive less than 200 nA at tens of volt, and therefore is only suitable for very low-current low-speed applications.

### Temporal Evolution of Charge Distribution, Self-Heating and Bias-Stress

The charge carrier mobility in organic semiconductors is known to improve with temperature. In addition, polymeric materials as used for plastic substrates are generally weak thermal conductors. For these reasons, gradual self-heating of organic devices at high I × V points has a large impact on the device characteristics.

As will be explained in the next section, the developed pulse-biasing setup can also accurately monitor the variations of V_BE_, i.e. ΔV_BE_ after applying a fixed I_E_ to the device. Measuring the temporal evolution of this ΔV_BE_ allows to study the effect of self-heating with the highest sensitivity, because in case of self-heating, this ΔV_BE_ would be proportional to the increase of the device temperature, i.e. ΔT, and therefore to the power dissipated in the device. This fact is because organic semiconductors have usually strongly increasing mobility with temperature^14,15^. Further, the contact resistance strongly decreases simultaneously^15^, resulting in a lower required V_BE_, i.e. negative ΔV_BE_, at fixed I_E_. This ΔV_BE_ would be linearly proportional to ΔT when ΔT is small, but in general it is a nonlinear function.

**Figure 3:**
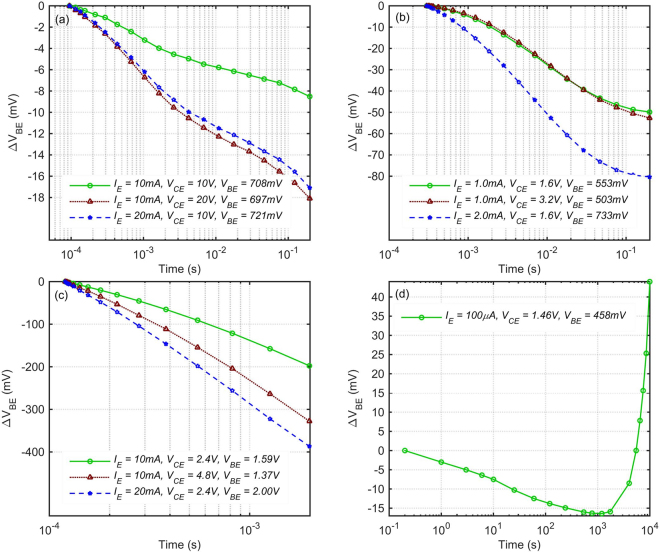
V_BE_ change due to self-heating, lateral diffusion of charge carriers and bias-stress. (**a**) Self-heating in a standard silicon bipolar junction transistor, 2N3904. (**b**) The OPBT at low-current, only showing lateral diffusion of charge carriers. (**c**) The OPBT at high-current, showing both self-heating and lateral diffusion of charge carriers. (**d**) Long-time bias-stress in the OPBT.

For the purposes of comparison and verification of this method, ΔV_BE_ of a general purpose, silicon Bipolar Junction Transistor (BJT) is measured at two different I_E_ × V_CE_ products of 100 mW and 200 mW, and is shown in Fig. 3(a). Conventional BJT is also a vertical device, but the silicon substrate conducts heat around 10 times better than the glass. In this experiment, tracking of the V_BE_ variation is started 90 μs after turning on the device. The V_BE_ variation during the initial 90 μs was smaller than the accuracy of the measurement setup. 200 mW is applied one time by doubling the current and one time by doubling the voltage. As expected, in both cases ΔV_BE_ is nearly two times that of the 100 mW. This confirms that here we only have self-heating, but no stress effects.

Similar experiments are performed on the OPBT at two low and high current levels as shown in Fig. 3(b and c). The V_BE_ and V_CE_ given in this figure are average values over the measurement time. In Fig. 3(b), although we have a considerable amount of ΔV_BE_ at 1 mA × 1.6 V, surprisingly, doubling the V_CE_ does not affect the ΔV_BE_, whereas doubling the current increases it by ~60%. This proves that in this case the observed effect is not self-heating.

As shown in Fig. 1(a), although the emitter window is 200 μm × 200 μm, the underneath base and collector electrodes are wider. Therefore we speculate that this effect is induced by the lateral diffusion of some of the electrons accumulated around the base oxide towards the outside of this window. This diffusion gradually increases the effective active area of the channel, and therefore decreases the required V_BE_ at the fixed I_E_ = 1 mA by the amount of ~9% after 200 ms in Fig. 3(b). Increasing the collector voltage has a small impact on the charge in the C_60_ layer of the emitter side, whereas increasing the I_E_ largely affects this charge density and the required V_BE_.

At a 15 times higher I_E_ × V_CE_ shown in Fig. 3(c), doubling the current increases the ΔV_BE_ ~95%, but here doubling the voltage also increases it ~66%. This indicates that in this case in addition to the lateral diffusion of the charge carriers, self-heating is also occurring and is important. Since self-heating has a considerable impact after hundreds of μs at 10 mA, obviously at 100 mA range it would already have an impact after tens of μs. In order to circumvent this effect, the pulse-biasing circuit developed here can turn on and apply an accurate bias to the device under test within few μs, and immediately start doing small-signal analysis afterwards.

Figure 3(d) shows a long-time DC stress measurement, performed using the Keysight B2912A Precision SMU, at a very low current of 100 μA at which self-heating would be negligible. The ΔV_BE_ tracking is started ~200 ms after turning on the device. Here, we clearly see different mechanisms occurring in different directions. The ΔV_BE_ initially goes negative due to the lateral charge diffusion, but finally starts increasing due to the bias-stress effect. Surprisingly, the lateral charge diffusion seems to be the dominant effect for an incredibly long time of ~1000 s. This might really be the case, because far away from the A_act_ there is no lateral electric field, and the undoped C_60_ layer could be extremely resistive causing very slow diffusion of electrons. However, other unknown mechanisms might also be the reason, such as very slow changes in the morphology of C_60_ or base-oxide under the electric field. We cannot provide a concrete explanation for the observed behavior. Anyway, it is a very slow and small effect in the mV range.

## Pulse-Biasing Small-Signal Characterization Circuit

**Figure 4:**
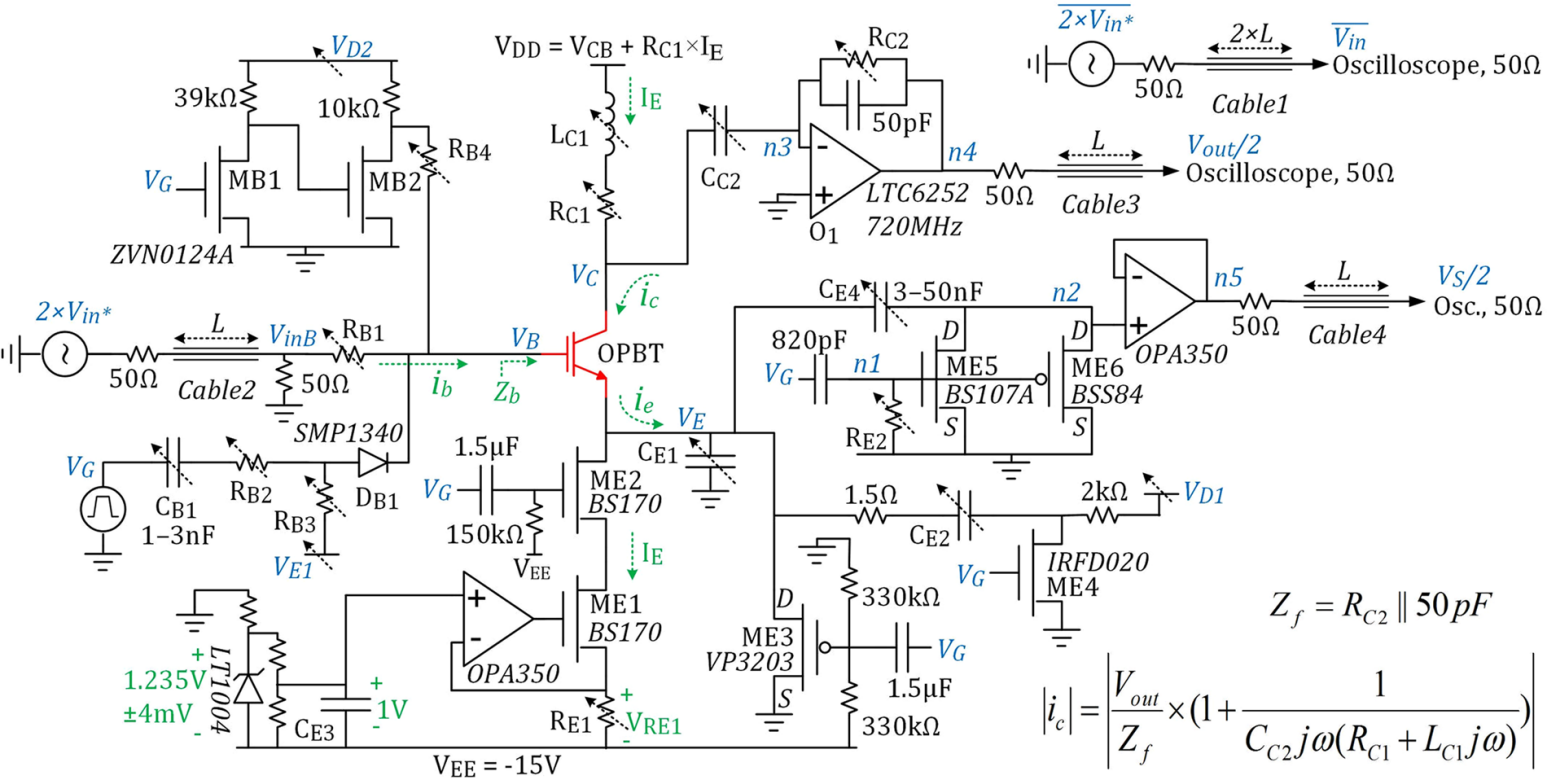
Simplified schematic of the new pulse-biasing small-signal measurement setup.

**Figure 5:**
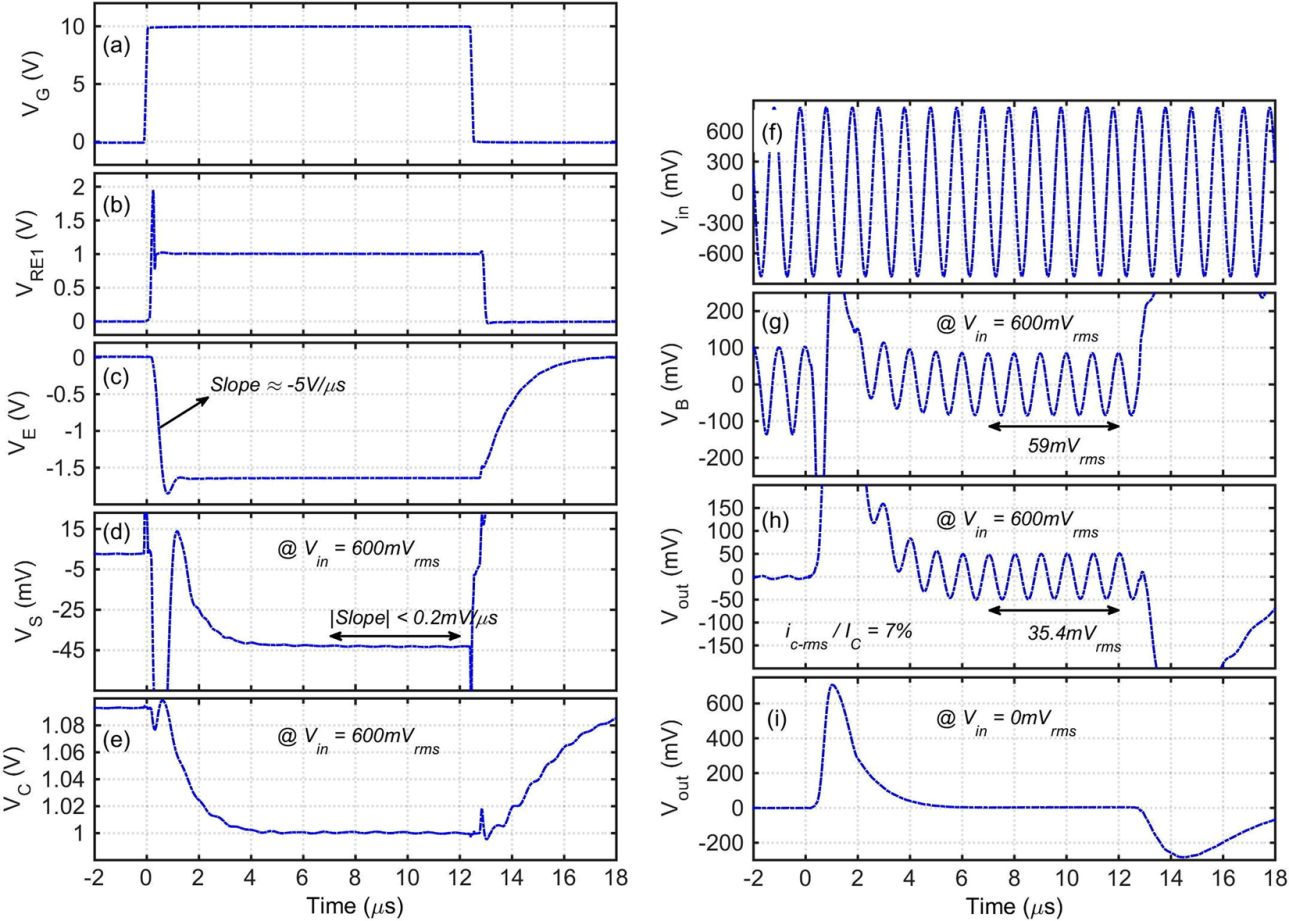
Signal waveforms of the characterization setup at pulse I_E_ = 10 mA and bias V_CB_ = 1 V, R_E1_ = 100 Ω, C_E1_ = 0.35 μF, C_E2_ = 0.1 μF, C_C2_ = 0.22 μF, L_C1_ = 5 μH, R_C2_ = 50 Ω, R_B1_ = 4.7 kΩ, R_B4_ = 1 MΩ and frequency = 1 MHz. (**a–h**) Signals are measured concurrently at V_in_ = 600 mV_rms_. (**i**) V_out_ is measured at V_in_ = 0 V_rms_.

Figure 4 shows the simplified schematic of the new measurement setup. Examples of the waveforms are illustrated in Fig. 5. An accurate 1 V reference is generated using the LT1004 voltage reference, and is used for defining the pulse I_E_ passing through R_E1_ = 1 V/I_E_ as shown in Fig. 5(b). DC voltage at the base electrode is zero. ME3 forces V_E_ = 0 V when there is no V_G_ pulse, keeping the OPBT off. The 10 V low-duty-cycle gating pulse V_G_ turns on ME2 on its rising edge to start the pulse bias current I_E_, and turns off ME3. The small-signal excitation comes from the sinusoidal source 2 × V_in*_, while C_E1_ and C_C2_ keep both emitter and collector at small-signal AC ground. Applying and stabilizing of the bias point at different nodes is done over the time interval of 0 to ~6 μs. Circuit parameters such as V_D1_, V_E1_ and R_C1_ are manually tuned to minimize this initialization time. The small-signal measurements are carried out afterwards.

At the base side, we have R_B4_ ≫ R_B1_ ≫ 50 Ω, and the input base impedance |Z_b_| ≈ 1/C_b_ω<<R_B1_ at the measurement frequency. The diode D_B1_ has less than 0.3 pF parasitic capacitance and is completely off during the small-signal measurement time.

### h_21_ and g_m_ Measurement Circuitry

The small-signal sinusoidal excitation comes from a differential signal generator. The invert of V_in_ arrives at the oscilloscope, and then V_in_ is calculated from it. The Cables 1–4 are 50 Ω, i.e. V_in_ and V_inB_ are slightly delayed versions of V_in*_. Since R_B1_ ≫ 50 Ω we have |V_inB_| ≈ |V_in*_| = |V_in_|. i_c_/i_b_/i_e_ are small-signal currents into the collector/base/emitter, respectively. As long as R_B1_ > 7/C_b_ω, |i_b_| can be estimated as |V_in_|/R_B1_ with less than 1% error. However, by measuring the signal amplitude at V_B_ using a high-impedance probe, as shown in Fig. 5(g), we can calculate the input base capacitance C_b_ and then calculate |i_b_| even more precisely.

At the collector side, the large-bandwidth op-amp O_1_ forces n3 to be ground and the large coupling capacitor C_C2_ keeps the collector to be AC ground. L_C1_ provides a path for the pulse I_E_ bias current, and L_C1_ω ≫ 1/C_C2_ω, i.e. most of the i_c_ passes through C_C2_, but C_C2_ is so large that the small-signal voltage amplitude at the collector is much smaller than the signal at the base. It is possible to calculate the exact value of |i_c_| using the equation given in Fig. 4. Since I_E_ is a pulse, it is important to make sure that L_C1_–C_C2_ do not cause additional ringing on the V_out_. This is assured by tuning R_C1_ ≈ 2 × sqrt(L_C1_/C_C2_). As shown in Fig. 5(i), when I_E_ pulse is applied but V_in_ is zero, no ringing appears on the V_out_ after Time = 6 μs. A 50 pF capacitor is added to the feedback loop around O_1_ to improve the stability.

Cable1 is intentionally twice the length of Cables 2 and 3; so that the signal delay from V_in*_ to V_inB_ plus the signal delay from n4 at the output of the op-amp O1 to V_out_ would be equal to the delay along Cable1. In this way, we could also measure the phase of h_21_ by measuring the phase difference between V_in_ and V_out_ on the oscilloscope. However, only the magnitude of h_21_ is needed to extract the f_T_, and |h_21_| = |i_c_|/|i_b_|.

At the emitter side, a very large C_E1_, in the μF range, is needed to keep the emitter at AC ground during the small-signal measurement after Time = 6 μs. On the other hand, a time much larger than T_0_ = V_BE_ × C_E1_/I_E_ would be needed for V_E_ to reach its required value; with the parameter values given in Fig. 5, this would be T_0_ = 57 μs. In order to significantly speed up this initialization time, the power switch ME4 is added. During the long off time, i.e. V_G_ = 0 V, C_E2_ is charged to a tunable voltage V_D1_ in the range of 0–30 V, and then on the rising edge of V_G_, a very large transient current up to V_D1_/1.5 Ω is sunk through C_E2_ and as shown in Fig. 5(c) this current quickly charges C_E1_ to a V_BE_ that enables an OPBT current exactly equal to the I_E_. However, this is true only if after this initialization time, no part of the I_E_ passes through C_E1,2,4_. In other words, the gradual slope of V_E_ should become zero.

To accurately monitor the gradual slope and variations of V_E_ after its sharp falling edge, V_G_ turns on ME5 for the first 1–3 μs, and during this time the small capacitor C_E4_ is charged to the initial emitter voltage. Then the kΩ resistor R_E2_ quickly pulls n1 to 0 V, turning off ME5, resulting in a >4 GΩ resistance across ME5,6. Because of the large time constant of C_E4_ × R_DS-ME5,6_, any further mV variations at V_E_ will be directly tracked at n2 and V_S_. V_D1_ is manually tuned to have ~0 mV/μs gradual slope at V_S_ over the small-signal measurement interval. Comparing Fig. 5(c to d), the V_S_ circuitry is providing ~25X zoom on the V_E_ for accurate tuning of V_D1_. ME6 just forces n2 to ground on the falling edge of V_G_.

On the falling edge of V_E_, a large R_B1_ × C_b_ time constant can slow down the charging of the base-emitter capacitance and increase the required initialization time. To make this faster, on the rising edge of V_G_, a certain amount of charge is injected into the base through C_B1_-R_B2_-D_B1_. V_E1_ is a negative DC voltage that controls the amount of this charge, and turns off the D_B1_ afterwards. V_E1_ is manually tuned to keep V_B_ around 0 V.

DC leakage current into the base, I_B_, is usually negligible. However, in case of a large I_B_, R_B4_ can be added, and V_D2_ controls the DC current injected into the base.

### Intrinsic Gain Measurement Method

For measuring the intrinsic gain, V_E_ is accurately measured at two slightly different collector voltages, but with equal pulse I_E_. Then we would have g_m_ × ΔV_BE_ = ΔV_CE_/r_out_. Therefore A_v0_ = g_m_ × r_out_ = ΔV_CE_/ΔV_BE_ is obtained. For this measurement V_B_ is grounded, C_E1,2_ are not needed, and V_C_ is shorted to V_DD_.

### Measurement Limits

The low-leakage BS170 MOSFET used as ME1,2 current source can drive up to ~300 mA into the OPBT. Larger MOSFETs with higher current drive capabilities, and accurate resistors down to 100 mΩ range for R_E1_ are widely available in the market. Therefore, accurate I_E_ pulses up to 10 A should be feasible with the proposed circuit, but it was not needed in our case study. At I_E_ < 1 μA range, the gate leakage through ME1,2 can cause bias inaccuracies; therefore ultra-low leakage MOSFETs would be needed for this case. The maximum bias voltages V_CB_ and V_BE_ are basically controlled by the V_DD_ and V_EE_; there is no specific limit in this regard.

The shortest measurement pulse width is mainly limited by the required initialization time for reaching the steady state (~6 μs in Fig. 5); afterwards, the small-signal information can be extracted in principle even from one sinusoidal signal cycle. As can be seen in Fig. 5(b), the I_E_ pulse settles in less than 2 μs. The charge injector circuitry ME4-C_E2_ has a very short R-C time constant of <160 ns. However, the OPBT capacitances and the frequency of the sinusoidal signal impose the main limitations on the initialization time. Although the charge injector path C_B1_-R_B2_-D_B1_ significantly speeds up the charging of the base capacitance C_b_, as can be seen in Fig. 5(g), still some time (~3 μs) proportional to R_B1_ × C_b_ is needed for the V_B_ to reach the steady state after D_B1_ turns off. On the collector side, C_C2_ should be more than 20 × 20 times larger than C_b_ to keep the small-signal voltage at V_C_ twenty times smaller than V_B_ at the frequency where |h_21_| = 20 is measured. We recommend L_C1_C_C2_ω^2^ > 10 so that the inaccuracy of the L-C components does not make large error in the i_c_ equation in Fig. 4. Taking all these points into account, practically we found that by optimizing the circuit parameters, the initialization time can be minimized to 5–7 periods of the sinusoidal signal.

## Further Discussions and Conclusion

The proposed pulse-biasing small-signal measurement circuit enables analog characterization of the device over >10 times wider I–V range comparing to the simple DC biasing approach^13,23^, and provides a much better control on the junction temperature by adjusting the pulse duration. It can also precisely track the ΔV_BE_ at different power densities for better understanding of the self-heating effects.

For the ~12 μs measurement shown in Fig. 5, if we repeat the pulse every 1.2 s, the waveforms can still be well monitored on the oscilloscope, while the bias-stress rate in the device decreases by a factor of ~10^5^ comparing to the DC biasing method. Therefore, many more measurements can be performed on the same fresh device during several days, providing reliable data for device modeling.

The OPBT currently suffers from a low charge carrier mobility in the vertical direction ~0.06 cm^2^/V.s^23^. However, the study presented in this work proved that despite this very low mobility, it can already reach the record f_T_ of 40 MHz and the intrinsic gain of 35 dB at V_CE_ = 8.6 V thanks to the short channel length. There is certainly a large room for further improvement of this speed by developing organic semiconductors which can provide higher mobility in the vertical direction. This can be better understood by considering the fact that in FETs, above the threshold region g_m_ and therefore f_T_ are proportional to sqrt(μ × I_E_). This g_m_ − I_E_ trend can be seen in Fig. 2(c), and more importantly, no g_m_ or f_T_ saturation is observed at high currents, confirming the room for reaching higher speeds. A higher mobility will also result in a lower bias voltage, power and self-heating for reaching the same I_E_. In planar organic FETs, mobility in the range of 3–30 cm^2^V^−1^s^−1^ has been reported by several groups^12,27–29^. Device simulations also predict sub-nano-second intrinsic switching delay for OPBTs with a mobility of 10 cm^2^/V.s^30^.

A f_T_ ≥ 40 MHz can pave the way for integrating new functionalities into organic circuits and systems. Long-distance wireless communication is the first example, because in this case the key challenge is the extremely large size of the antenna required to have electromagnetic wave radiation in MHz regime. For example, an electrically-small single-turn loop antenna has a radiation resistance, and therefore efficiency, roughly proportional to *frequency*^4^ × *D*^4^, where *D* is the diameter of the loop; an antenna with D = 1 m radiates with an efficiency of 18% at 10.1 MHz^31^. Therefore, we can expect ~7% RF power radiation at 40 MHz with D = 20 cm which is a reasonable size for integration onto human body or cloth, or toys. Wireless communication can also be done in pulse mode. For example, super regenerative receivers, which are in use since 1940s^32^, turn on an oscillator just for a few μs, receive the data, and then turn it off again for a long time, similar to how we operated the device in this work.

As the second example, inductor-based switching power converters can be mentioned. Power supply is an essential part of any electronic system, and inductor-based switching power converters are the most energy efficient circuits widely used in up/down DC–DC converters and battery chargers. Key elements of such circuits are FET switches, diodes, inductors and capacitors. A typical switching circuit working with <1 μs pulses would require spiral inductors in the 10 μH range which can be easily fabricated by inkjet printing or evaporating a single metal layer on a plastic substrate with outer diameter <10 cm. Organic rectifying diodes already can work at tens of MHz^33,34^. Printed flexible 10 μF range multi-layer capacitors for power conversion applications have been demonstrated^35^ and show a high relative dielectric constant of 15–22 up to 10 MHz. In addition to the high f_T_, the high current drive capability of the OPBT is also an advantage for compatibility with this application. In this context, it also worth to mention that flexible batteries and organic solar cells are also already developed and even commercialized.

The pulse measurement results are also relevant for digital logic circuits in which we only have transient currents in the transistors.

## Methods

The OPBT presented here is built in a single chamber UHV-tool. The glass substrate is previously cleaned with N-Methylpyrrolidone, distilled water, ethanol, and an Ultra Violet Ozone Cleaning System. By using thermal vapor deposition at high vacuum conditions (p < 10^−7^ mbar), the OPBT stack layers are deposited through laser-cut, stainless steel shadow masks. The layer stacks, evaporation rates, and treatments are: Al 100 nm (1.0 Å/s); Cr 10 nm (0.1 Å/s); i-C_60_ 100 nm (1.0 Å/s); Al 15 nm (1 Å/s); 15 min oxidation at air; i-C_60_ 100 nm; 2 times (perpendicular to each other) SiO 100 nm with a free stripe of 200 μm (1.0 Å/s); n-C_60_ 20 nm (0.4 Å/s) co-evaporating C_60_ with W_2_(hpp)_4_ (purchased from Novaled GmbH, Dresden) with 1 wt%; Cr 10 nm (0.1 Å/s); Al 100 nm (1.0 Å/s); encapsulation in a nitrogen atmosphere using UV cured epoxy glue without UV exposure on the active area; annealing for 2 h at 150 °C in a nitrogen glove-box on a heat plate, for positively affecting both current-transmission through the base and regeneration of the air-exposed C_60_^36,37^.

The 50 pF capacitor across the op-amp O_1_ is a mica capacitor, but other capacitors in the circuit are polypropylene or polyester film capacitors. For C_E1,2,4_ polypropylene is preferred.

Figure 1(a) has been taken using the Nikon microscope ECLIPSE LV 100 ND. Dot lines are added around the base electrode and the active area. This photo is not recolored; base and collector look colorful because of the materials deposited on top of them; emitter electrode looks white because it is on the top.

### Data availability

All data generated or analysed during this study are included in this published article.
